# Respective contribution of the cephalic neural crest and mesoderm to SIX1-expressing head territories in the avian embryo

**DOI:** 10.1186/s12861-017-0155-z

**Published:** 2017-10-10

**Authors:** Barbara F. Fonseca, Gérard Couly, Elisabeth Dupin

**Affiliations:** 1 Sorbonne Universités, UPMC Univ Paris 06, INSERM, CNRS, Institut de la Vision, 17 rue Moreau, 75012 Paris, France; 2 Université Paris Descartes, Institut de la Bouche et du Visage de l’Enfant, Hôpital Universitaire Necker, 149, rue de Sèvres, 75015 Paris, France

**Keywords:** SIX1, Neural crest, Mesoderm, Quail-chick chimera, Branchial arch

## Abstract

**Background:**

Vertebrate head development depends on a series of interactions between many cell populations of distinct embryological origins. Cranial mesenchymal tissues have a dual embryonic source: - the neural crest (NC), which generates most of craniofacial skeleton, dermis, pericytes, fat cells, and tenocytes; and - the mesoderm, which yields muscles, blood vessel endothelia and some posterior cranial bones. The molecular players that orchestrate co-development of cephalic NC and mesodermal cells to properly construct the head of vertebrates remain poorly understood. In this regard, *Six1* gene, a vertebrate homolog of *Drosophila Sine Oculis*, is known to be required for development of ear, nose, tongue and cranial skeleton. However, the embryonic origin and fate of *Six1*-expressing cells have remained unclear. In this work, we addressed these issues in the avian embryo model by using quail-chick chimeras, cephalic NC cultures and immunostaining for SIX1.

**Results:**

Our data show that, at early NC migration stages, SIX1 is expressed by mesodermal cells but excluded from the NC cells (NCC). Then, SIX1 becomes widely expressed in NCC that colonize the pre-otic mesenchyme. In contrast, in the branchial arches (BAs), SIX1 is present only in mesodermal cells that give rise to jaw muscles. At later developmental stages, the distribution of SIX1-expressing cells in mesoderm-derived tissues is consistent with a possible role of this factor in the myogenic program of all types of head muscles, including pharyngeal, extraocular and tongue muscles. In NC derivatives, SIX1 is notably expressed in perichondrium and chondrocytes of the nasal septum and in the sclera, although other facial cartilages such as Meckel’s were negative at the stages considered. Moreover, in cephalic NC cultures, chondrocytes and myofibroblasts, not the neural and melanocytic cells express SIX1.

**Conclusion:**

The present results point to a dynamic tissue-specific expression of SIX1 in a variety of cephalic NC- and mesoderm-derived cell types and tissues, opening the way for further analysis of *Six1* function in the coordinated development of these two cellular populations during vertebrate head formation.

**Electronic supplementary material:**

The online version of this article (10.1186/s12861-017-0155-z) contains supplementary material, which is available to authorized users.

## Background

The head of vertebrates is built during development through the growth and differentiation of specialized structures and cell types which derive from distinct embryological primordia: the neural plate, yielding the brain while the ectodermal placodes are at the origin of most of the cephalic sense organs, in collaboration with the cells migrating from the neural crest (NC) to form cranial sensory ganglia. Besides the CNS and peripheral nervous system (PNS), most tissues in the head of amniote vertebrates develop from mesenchymal progenitors that ensure the production of the cranial dermis, bones, cartilages and blood vessels as well as tendons, muscles, fat and connective tissues. These mesenchymal cranial tissues are derived from two main embryological sources, the mesoderm and the NC, whereas, in the trunk, they have a unique mesodermal origin. It is well recognized that most of the cephalic mesenchyme arises from the NC. As theorized by Gans and Northcutt in a seminal article published in 1983, the “new head” that vertebrates acquired during evolution is mainly due to the production of NC derivatives rostral to the notochord [1]. Thus, the NC cells (NCC) provided a skull and facial tissues to accompany the expansion of the prosencephalon and, in cooperation with ectodermal placodes, they participated in the addition of sophisticated sensory modalities, resulting in the complex head structures and brain, which allowed successful radiation of modern vertebrates.

Regarding head skeletogenesis, a triple origin of the craniofacial skeleton has been defined in the avian embryo thanks to quail-chick chimera experiments [2]; thus, the head skeleton is formed by concerted development of the anteriormost NC mostly yielding facial structures, the cranial paraxial mesoderm and, for its caudalmost part, the somitic mesoderm [3]. How the various bone and cartilage rudiments arising from mesodermal and NC precursors develop in concert to form the head skeleton, and how they assemble with muscles and tendons for example, are main issues in craniofacial biology. The establishment of the vertebrate head vascularization represents another striking example of the cooperation between the developing NCC and mesodermal cells: the cephalic vascular system is established from both the mesoderm, − for the endothelia of blood vessels - and the NC, − for the smooth muscle cells and pericytes lining these endothelia [4, 5]. It is therefore clear that the building of most of the distinct mesenchymal tissues of the head depends on tightly coordinated interactions between the cranial NC and mesoderm.

Molecular regulation of commitment and differentiation of mesenchymal tissues is mediated in part, by the action of transcription factors that control the downstream activity of the genes encoding the specialized proteins needed for the generation of the distinct types of differentiated mesenchymal cells. Among these transcription factors, the *Six* homeobox family of genes are vertebrate homologs of genes required for eye development in *Drosophila* [6]. Three subgroups of *Six* genes have been characterized in vertebrates, including *Six1/Six2*, homologs to *Drosophila Sine Oculis*, *Six3/Six6* homologs to *Optix* and *Six4/Six5* homologs of *Dsix4* [7]. In the mouse, while *Six3* mainly functions in eye and rostral CNS development [8–11], differential expression and effects of the other *Six* genes have been described in a number of tissues distinct from the brain, such as muscles, kidney, the auditory system, genitalia, several sensory organs and craniofacial structures [7]. *Six1* is well known as a pan-placodal marker, labeling the pre-placodal domain at presomitic stages and, later on, all the placodes (except the lens) and most of their derivatives [12, 13]. With respect to the development of mesenchymal tissues in the mouse, *Six1* and *Six2* are expressed in the developing limb and somitic mesenchyme [14–16], as well as in the head and branchial arch (BA) mesenchyme, and in kidney nephrogenic chords [17]. *Six1* is required for early steps of myogenesis [15, 18]. Its inactivation in mice also causes defects in kidney, thymus, inner ear and craniofacial development [17]. In human, *SIX1* gene haploinsufficiency results in the branchio-oto-renal syndrome, an autosomal dominant developmental disorder characterized by hearing loss and branchial defects [19, 20] while defects in *SIX2* cause conductive hearing loss [21] and mild forms of frontal dysplasia [22].

Loss of function of *Six* genes in the mouse triggers several notable effects on cranial NC development. *Six1* mutant fetuses exhibit shorter squamosal, mandibular and maxillary bones, Meckel’s cartilage and hyoid bone, which are formed by NCC of BA1, BA2 and BA3 [17]. In contrast, invalidation of *Six2* specifically affects the formation of the endochondral skeleton of the cranial base, including the basisphenoid bone [23], which has a dual origin, from the NC, anteriorly, and from the cranial mesoderm, in its posterior part [2, 24–26]. *Six2*-null newborn mice display premature bone fusion due to abnormal chondrocyte differentiation, although initial migration and skeletogenic differentiation of NCC appeared unaffected [23]. In mouse BA2 NCC, *Six2* has been shown to be a direct target of *Hoxa2*, the most anteriorly expressed *Hox* gene, which is present in BA2 and represses *Six2* [27, 28]. The more rostral NC (from mid-diencephalon to rhombomere-2 included), is endowed with a characteristic *Hox*-free status that is required for the formation of the NC-derived facial skeleton, and, indirectly, for anterior brain development [29–32]. In the chick embryo, Garcez et al. (2014) have reported that the silencing of *SIX2, SIX1* and *SIX4* genes in the early NC, by *in ovo* electroporation of double-strand RNAs, results in severe hypoplasia of the facial skeleton and atrophy of the anterior dorsal brain [33]. Moreover, when the 3 *SIX* genes are silenced simultaneously, the skeletal and brain defects are exaggerated and phenocopy those obtained after ectopic expression of *HOXA2* in the premigratory cephalic NC [31]. According to rescue experiments, these authors concluded that *SIX* genes, expressed in the anterior cephalic NCC from the migratory stage, can be negatively regulated by *HOXA2* and likely function to control proliferation and cell death in the developing cranial NC mesenchyme [33].

Taken together, the data described above argue that, among pleiotropic expression and effects on the vertebrate embryo, *Six1*/*Six2* genes are crucial for the development of head mesenchymal tissues, although their precise dynamics of expression and their respective role in the NC and mesodermal cells remain unclear. In order to address this issue, we have used the avian embryo model to investigate the detailed spatial-temporal pattern of expression of *SIX1* gene and SIX1 protein, particularly regarding the fate of mesoderm- and NC-derived cells in the developing head. By using in vivo quail-chick transplantations of the NC and mesoderm as well as in vitro cranial NCC cultures, the present study highlights the respective contribution of NC and mesodermal cells to the deployment and differentiation of SIX1-expressing cells in head mesenchymal tissues.

## Methods

### Chicken embryos handling and cryosectioning

Fertilized eggs of *Gallus gallus* chicken were obtained from a commercial source (EARL Les Bruyères, France) and incubated at 38.5 °C in humidified conditions. Embryos were collected at different developmental time points. Stage determination was done according to Hamburger and Hamilton (1951) (HH stage) [34] and, for embryos until 2 days of incubation (E2), by counting the number of somite pairs, here referred to as somite-stage (ss). Embryos were fixed in 4% formaldehyde for 2 h at room temperature or overnight at 4 °C, washed in PBS, embedded in 30% sucrose overnight, and frozen in isopentane at −50 °C (temperature stabilized with dry ice). Frozen sections were cut at 18 μm with a cryostat (Leica).

### Construction of quail-chick chimeras of the cranial NC and mesoderm

Quail (*Coturnix Coturnix japonica*) and chicken (*Gallus gallus)* fertilized eggs were obtained from commercial sources (Cailles de Chanteloup and EARL Les Bruyères, France) and incubated at 38.5 °C in humidified conditions for about 30 h in order to obtain embryos at stage HH8 (5ss). The NC chimeras were constructed by isotopic and isochronic replacement of the chick cranial neural fold (i.e. the premigratory NC) by its quail counterpart, according to previous experiments of NCC fate mapping [2, 35]. The graft of neural fold encompassed the rostrocaudal level from the midbrain to anterior rhombencephalon. Cranial mesoderm quail-chick chimeras were prepared as previously described [4, 36]: a portion of the quail paraxial cephalic mesoderm was microsurgically isolated after opening of the superficial ectoderm lateral to the mesencephalon at stage HH8 (5ss); the excised cranial mesoderm was then grafted in an identical position in a stage-matched chick host embryo. All the quail to chicken grafts were performed unilaterally. Twenty-four hours after surgery, the host embryos (of stage HH15) were sacrificed and handled for cryosectioning as described above.

### In situ hybridization and immunohistochemistry on sections

Chicken *SIX1* riboprobes were prepared from a cDNA plasmid encoding full-length coding sequence of chicken *SIX1* (GenBank AB199734.1) (gift from Dr. Atsushi Kuroiwa; Nagoya University). The *SIX1* template was linearized with HindIII endonuclease and transcribed with a T3 polymerase in the presence of digoxigenin-11-D-UTP (Roche Diagnostics) to synthesize the antisense riboprobe. In situ hybridization on frozen sections was performed as previously described [37]. Briefly, after proteinase K treatment (10 μg/mL, Invitrogen) and postfixation in 4% PFA, slides were pre-hybridized for 2 h at room temperature in hybridization buffer (50% formamide, SSC 5X, Denhardt’s 1X, 50 μg/mL yeast tRNA and 500 μg/mL salmon testes DNA, pH 7.4; all reagents from Sigma). Hybridization took place overnight at 72 °C, in the same buffer, with *SIX1* riboprobe diluted at 1:200. Detection of the hybridization involved anti-DIG-AP antibody (1:5000, Roche Diagnostics) followed by color substrate reaction with nitroblue tetrazolium chloride (337.5 μg/mL) and 5-bromo-4-chloro-3-indolyl phosphate (175 μg/mL) (Roche Diagnostics). Cryosections were mounted in Mowiol (Calbiochem Darmstadt, Germany).

For the immunostaining, tissue cryosections were permeabilized for 1 h in PBS containing 5% fetal bovine serum, 1% bovine serum albumin and 0.1% Triton X-100, before overnight incubation with the following primary antibodies: anti-SIX1 (1:200; Sigma, HPA001893), anti-SOX10 (1:250; Santa Cruz, sc-17,342), anti-SOX9 (1:500; Millipore, AB5535) anti-chondroitin sulfate (CS) (1:1600; Sigma, C8035), anti-βIII tubulin TUJ1 (1:500; Covance, MMS435P). Supernatants from mouse hybridoma against QCPN (quail, non chick perinuclear marker) and MF20 (chicken myosin heavy chain) were purchased from DSHB (Developmental Studies Hybridoma Bank, University of Iowa, Iowa City, IA) and used undiluted. HNK1 antigen labeling was performed using 1:3 diluted supernatants from cultured hybridoma cells (HNK1, ATCC TIB-200). All secondary antibodies used were Alexa Fluor 488, 546 and 647-conjugated antibodies (ThermoFisher Scientific). Cryosections were counterstained with 4′,6-diamidino-2-phenylindole (DAPI) (10 mg/mL; Sigma) and mounted in Mowiol (Calbiochem Darmstadt, Germany). Analysis and imaging were performed with a fluorescence microscope (DM6000, Leica) coupled to a CoolSnapHQ camera (Roper Scientific) or whole section images were captured with a Nanozoomer 2.0 slide scanner (Hamamatsu).

### Quail cephalic NC culture and immunostaining

Quail cephalic NCC were obtained from 6 to 7 ss embryos (equivalent to stage HH9) and cultured essentially as previously described [38, 39]. Briefly, the neural primordium at the level of midbrain-anterior rhombencephalon, which includes the premigratory NC at this early stage, was isolated and plated in explant culture for 15–18 h; during this in vitro period, the NCC exit from the dorsal neural primordium and migrate on the substrate, similar to their behavior in vivo [40]. Approximately 1000-to-2000 of NCC per neural tube fragment thus can be harvested in such migratory cell outgrowth (after discarding the neural explant that remained epithelial) [38, 39]. Cranial NCC were then seeded on a 3T3 fibroblast feeder layer in 96-well plates (400 NCC per well) in DMEM containing 10% FCS and 2% chicken embryo extract (80 ng/mL). Cultures were maintained at 37 °C in a humidified 5% CO_2_ incubator and the medium changed every 3 days. After 6 days of culture, the cells were fixed in 4% formaldehyde for 30 min and then immunolabeled with anti-SIX1, anti-chondroitin sulfate (CS) and HNK1 antibodies, as detailed above for cryosections. Cultures were also analyzed using an antibody to α-smooth muscle actin (αSMA clone 1A4; 1:800 Sigma, A5228) and mouse anti-quail tyrosine hydroxylase hybridoma (undiluted supernatant) [41]. The cultures were counterstained with 4′, 6-diamidino-2-phenylindole (DAPI) to mark cell nuclei, which led quail cephalic NCC to be easily distinguished from mouse 3T3 fibroblasts by their distinct nuclear sizes [42]. Imaging and automatic quantification of labeled fluorescent cells in the cultures were carried out with an Arrayscan High-Content system (Thermo Fisher Scientific).

## Results

### Expression pattern of *SIX1* gene and SIX1 protein at early stages of avian cranial development

*SIX1* early expression pattern in the chick has been previously described [43, 44]: *SIX1* transcripts were detected as soon as stage HH8 in the cranial mesenchyme and otic vesicle, and later on, became widespread in the cranial mesenchyme. A recent report indicated that *SIX1*, as well as *SIX2* and *SIX4* genes, are expressed in the craniofacial NCC population at 10 ss (HH10) in chicken embryos [33]. However, at similar NC early migratory stages, Sato et al. (2012) found no overlap between GFP reporter expression, driven by *SIX1* enhancers electroporated in the chick, and immunoreactivity to the NC marker HNK1, thus conflicting with the results from in situ hybridization in whole chicken embryos reported before [33, 44]. The precise origin of SIX1-positive mesenchymal cells, from the cranial NC or the mesoderm, therefore, remains unclear.

**Fig. 1:**
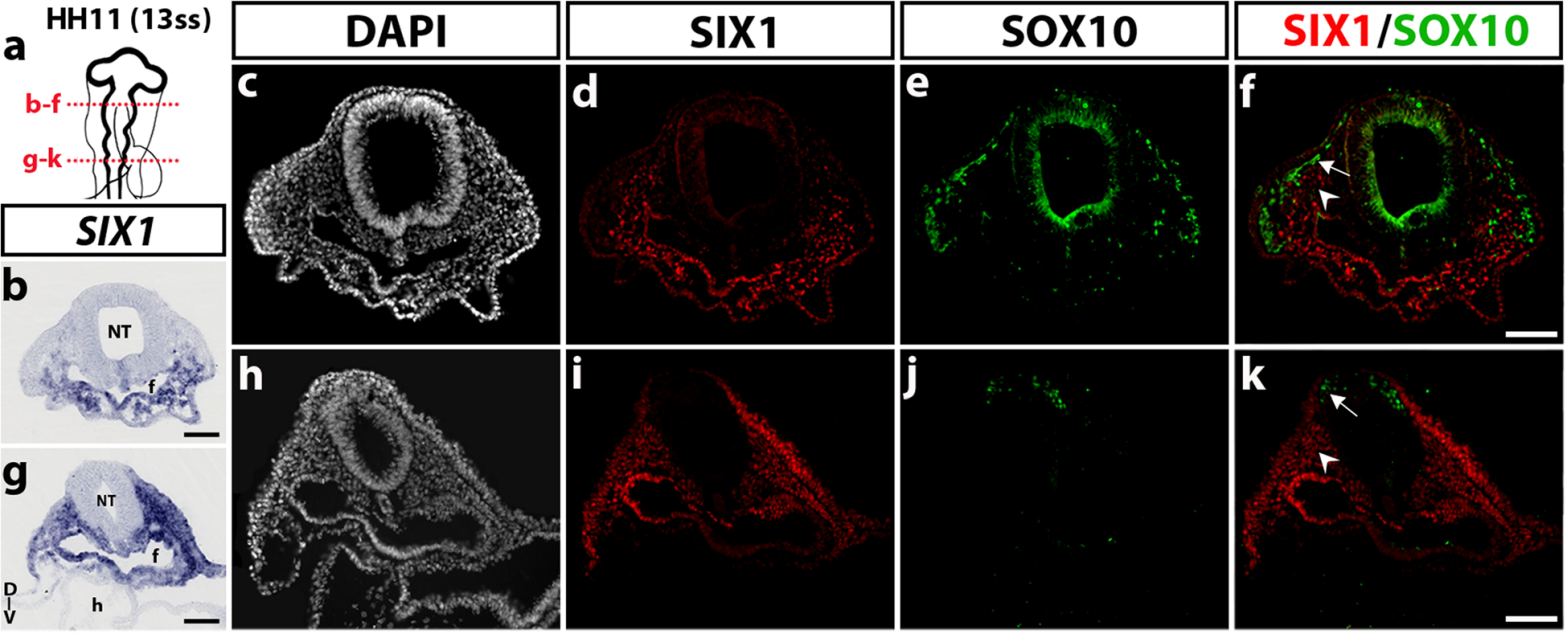
SIX1 expression pattern in the head of HH11 (13ss) chicken embryo. **a** Schematic representation of a HH11 (13ss) chicken embryonic head; red dotted lines indicate the position and orientation of the sections shown in **b**-**f** (mesencephalic level) and **g**-**k** (rhombencephalic level). **b** and **g** *SIX1* gene transcripts are detected in the foregut endoderm and in the cranial mesenchyme, located ventral to the mesencephalon (in **b**) and more laterally, at the rhombencephalic level (in **g**). **c**-**f** Transversal section, showing immunoreactivity to SIX1 (**d**) and SOX10 (**e**), with no apparent overlap (See merge staining in **f**). **d** and **i** Adjacent sections to **b** and **g**, respectively, after SIX1 immunostaining, show an expression pattern of SIX1 similar to that observed after in situ hybridization. **e** and **j** SOX10 antibody labels the NCC, which migrate from the mesencephalon dorsolaterally under the ectoderm in **e** and are starting to migrate from the rhombencephalon in **j**. **f** and **k** Merge of SIX1 and SOX10 stainings (arrows: migratory NCC; arrowheads: cranial paraxial mesodermal cells). D-V, dorso-ventral orientation; f, foregut; nt, neural tube; h, heart. Scale bars, 100 μm

In order to better define the tissue-specific expression of SIX1 transcription factor in the developing embryonic head, we first analyzed the distribution of *SIX1* mRNA and SIX1 protein in sections of chicken embryos of stage HH11, when migration of cranial NCC takes place (Fig. 1). Delamination of NCC from the dorsal most region of the neural primordium begins at the level of posterior diencephalon-mesencephalon then progresses caudally [35]. In HH11 (13ss) embryos (Fig. 1a), *SIX1* transcripts were detected in the mesenchyme ventral to the mesencephalon and in the anterior foregut endoderm while no transcripts were found more dorsally, near the neural primordium (Fig. 1b). A similar, ventral expression pattern was obtained after SIX1 immunostaining (Fig. 1d). Immunolabeling with SOX10, a recognized marker of NCC at this early developmental stage [45], marked the NCC migrating under the ectoderm towards the pharyngeal regions and did not overlap with SIX1 expression (Fig. 1 d-f). At the rhombencephalic level, both *SIX1* transcripts (Fig. 1g) and SIX1 immunoreactivity (Fig. 1i) were strongly detected laterally and ventrally to the neural primordium, in the non-neural ectoderm, the mesenchyme and the foregut endoderm. As shown in Fig. 1j-k, SOX10-positive NCC that started to migrate from the dorsal rhombencephalon did not express SIX1.

**Fig. 2:**
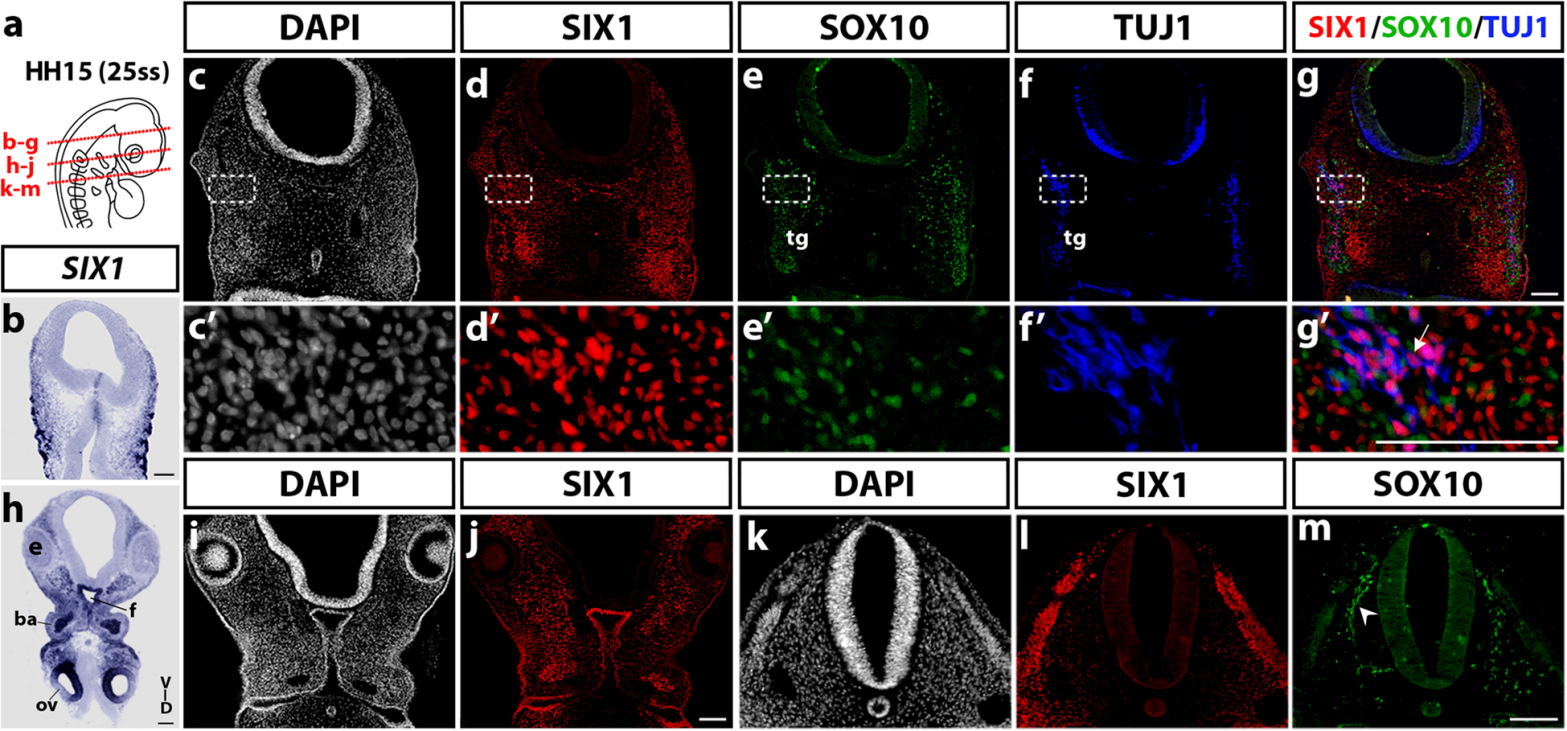
SIX1 expression pattern in the head of HH15 (25ss) chicken embryo. **a** Schematic representation of a HH15 chicken embryo; red dotted lines indicate the position and orientation of sections shown in **b**-**g**, **h**-**j** and **k**-**m** images. **b** and **h** *SIX1* transcripts expression in the lateral mesenchyme of the anterior head, and, in a more caudal section (**h**), in the periocular mesenchyme, foregut (f), branchial arch (ba) mesenchyme and superficial ectoderm and otic vesicles (ov) (e, eye). **c**-**g** Horizontal section (D-V dorso-ventral orientation); SIX1, SOX10 and TUJ1 immunostainings (the box area is magnified in **c’**-**g’**): SIX1 is expressed in the mesenchyme surrounding the trigeminal ganglion (tg) and in the trigeminal ganglion and nerves, where it labels a subset of TUJ1-positive neurons, not SOX10-positive non-neuronal cells (**c’**-**g’**); arrow in **g’** indicates a SIX1-positive neuron. **i**, **j** section at the level of the eye, showing SIX1 expression in the periocular cranial mesenchyme, the BA mesenchyme and the endoderm lining the pouch, **k**-**m** Section at the anterior somitic level; SIX1 is strongly expressed in the dermomyotome (**l**) while SOX10 labels the migratory NCC, indicated by an arrowhead (**m**). Scale bars, 100 μm

We further investigated SIX1 expression at a later stage, when the NCC have spread into the growing head and the BAs and become intermingled with paraxial mesodermal cells [46]. In the head of E2.5 chick embryos, at HH15 stage, *SIX1* mRNA-expressing cells were distributed under the ectoderm lateral to the mesencephalon (Fig. 2a, b), where SIX1-immunoreative cell nuclei were also detected, in the vicinity and within the forming trigeminal ganglion (Fig. 2c-d and *c’*-*d’*); the latter ganglion contained SOX10-positive non-neuronal cells of NC origin and TUJ1-immunoreactive neurons (Fig. 2 e-f and *e’* *-* *f’*). We did not observe colocalization of SIX1-positive and SOX10-positive nuclei, while SIX1 was co-expressed with TUJ1 in a subset of ganglion neurons (Fig. 2g and *g’*). This result is in agreement with recent findings showing that the SIX1-positive cells in the trigeminal ganglia are sensory neurons, which could belong to those of placodal origin in the mouse and chick embryos [44, 47]. At the level of the anterior rhombencephalon, *SIX1* transcripts were highly expressed in otic vesicles, pharynx and the periocular and pharyngeal mesenchyme (Fig. 2h). Interestingly, nuclei labeled with SIX1 antibody occupied the core of BA2, a region that later differentiates into jaw muscles (Fig. 2j). In a more posterior section of the same embryo, at the level of the first somites, SIX1-positive nuclei were detected in the dermomyotome whereas SOX10 antibody stained the NCC migrating ventrally to the cardiac region (Fig. 2l and m).

Taken together, these results indicate that the early expression pattern of SIX1 in the chick embryonic head varies with time and is not restricted to only one developmental source (NC, placode or mesoderm), neither to a unique fate (mesenchymal or neural). Of note, however, at early NC migratory stages (HH11), SIX1 expression appears excluded from the cranial NCC while broadly distributed in non-neural tissues, particularly the cranial mesenchyme.

### Cephalic NC and mesoderm origins of SIX1 expression domains, analyzed in avian quail-chick chimeras

Our data (Fig. 1) suggested a complex and dynamic expression of SIX1 during early cranial development in the chick embryo, when NCC migration and deployment of mesodermal cells led to their mixing and co-organization in the forming head. In an attempt to clarify the distribution of SIX1 transcription factor in cells derived from the cephalic NC and paraxial mesoderm in the early mesenchymal tissues, we have performed quail-chick transplantations *in ovo*, of either the cephalic NC or the cranial paraxial mesoderm. According to previous work by Couly and collaborators [3, 40], such transplantations allowed to establish the precise fate map of these two cell populations in the avian model.

**Fig. 3:**
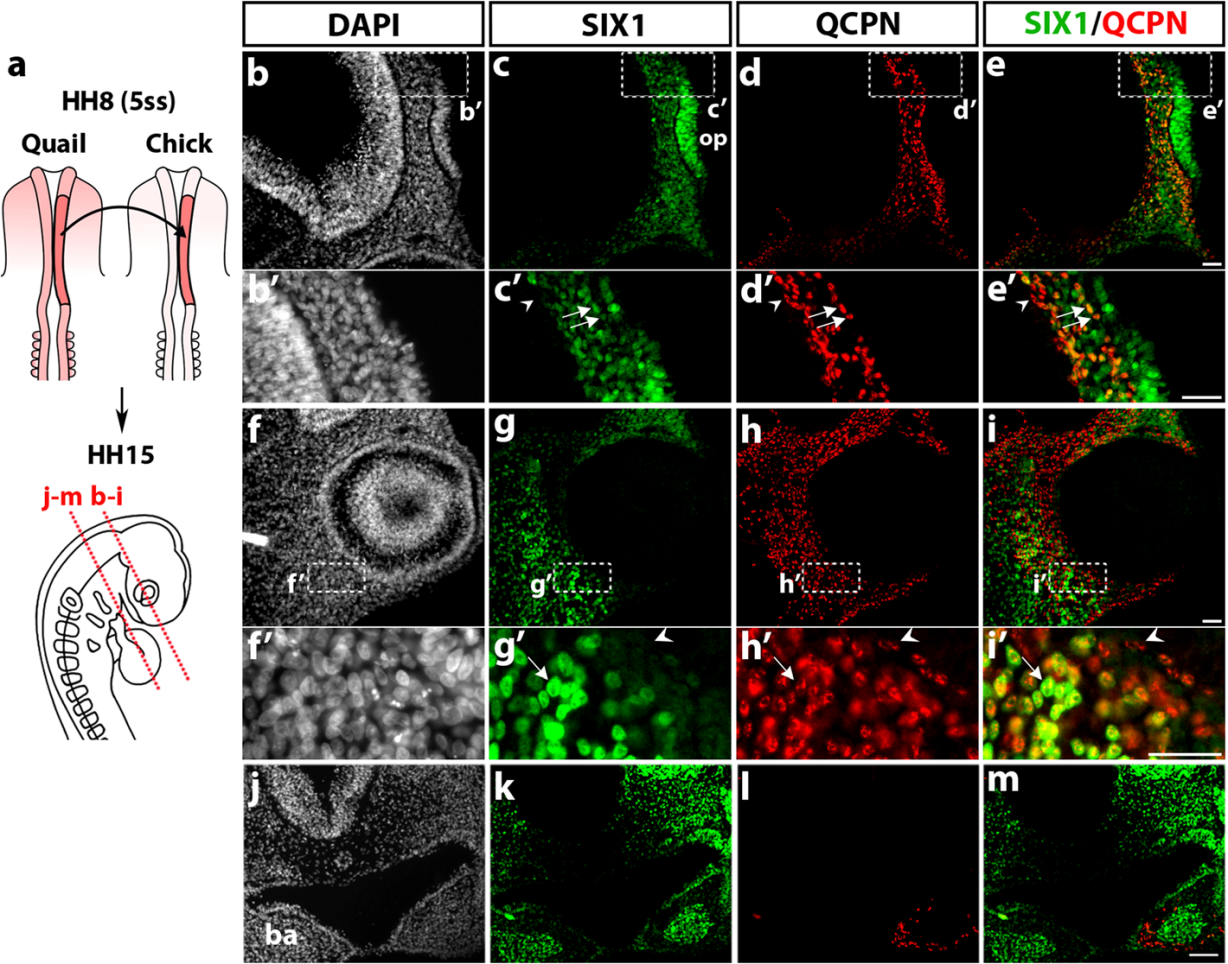
SIX1 expression in quail-chick chimeras of the cephalic NC. **a** Schematic representation of the cranial NC grafting procedure. The chick neural fold/premigratory NC from midbrain to anterior rhombencephalic level was unilaterally replaced by its quail counterpart (in red) at stage HH8 (5ss); the resulting chimeras were analyzed at HH15 (25ss) for SIX1 immunoreactivity, nuclear staining (DAPI) and the presence of QCPN+ quail cells (red dotted lines indicate orientation and position of the sections shown in **b**-**i** and **j**-**m**). **b**-**e** In the prosencephalic region, SIX1 (**c**) is expressed in the olfactory placode (op) and the adjacent cranial mesenchyme surrounding the prosencephalon, in which quail NCC, positive for QCPN are located (**d**); **b’**-**e’** Highlighting of the area boxed in **b**-**e**, showing engrafted quail cells that are either positive (arrows) or negative (arrowhead) for SIX1. **f**-**i** SIX1 and QCPN are both expressed in the head mesenchyme around the eye. **f’**-**i’** (magnified region indicated by a boxed area in **f**-**i**) Some NCC engrafted in the periocular mesenchyme co-stain with SIX1 and QCPN (arrow), while others do not express SIX1 protein (arrowhead). **j**-**m** In the maxillary region, SIX1 expression (**k**) is present in the mesenchyme dorsal to the foregut, in the foregut endoderm and the core region of BA1 (ba in **j**) whereas the grafted QCPN+ cells (**l**) are located in the peripheral mesenchyme of the BA and do not express SIX1 (**m** Merge of SIX1 and QCPN). Scale bars, 25 μm (**b**-**i**) and (**b’**-**i’**) and 50 μm (**j**-**m**)

The first type of transplantation involved the isotopic grafting of the premigratory cephalic NC, taken from a quail embryo at 5ss (stage HH8), into a chick host embryo of the same stage (Fig. 3a), as published previously [2, 48]. Analysis of quail-chick chimeras was performed approximately 24–30 h after the graft at stage HH15 (25ss), using both the QCPN antibody, to identify the grafted quail cells, and immunostaining for SIX1. Of note, control quail embryos at a stage equivalent to HH15 exhibited an expression pattern of SIX1 similar to the one observed in the chicken embryos (Additional file 1: Figure S1). In the chimeric embryos, sections of the anterior head showed the presence of numerous SIX1-positive cells in the presumptive nasal region (Fig. 3b, c), both in the olfactory placode and in the underlying mesenchyme near the prosencephalon; the grafted quail NCC populated this mesenchymal area (Fig. 3d, *d’*) and, in many cases, coexpressed SIX1 (Fig. 3e, *e’*). In addition, the periocular mesenchyme (Fig. 3f-i) comprised a high density of SIX1 and QCPN-immunoreactive cells; colocalization of these markers revealed that a subset of the engrafted NCC, homing towards the eye, expressed SIX1 (Fig. 3 *g’*-*i’*). More caudally (Fig. 3j-m), SIX1 labeling was found in the BAs, as already described (Fig. 2h, j), in the pharyngeal endoderm and mesenchyme, where SIX1-positive nuclei were clustered in the center of the BA2 (Fig. 3k). In contrast, the QCPN-positive NCC settled in the periphery of the BA mesenchyme and were distinct from SIX1-expressing chicken cells in the arch core (Fig. 3l, m). These data, therefore, showed that at least a subpopulation of the grafted cranial NCC, which populated the nasal and periocular mesenchyme, expressed SIX1, while SIX1 appeared excluded from the mesenchymal NCC in the BAs.

**Fig. 4:**
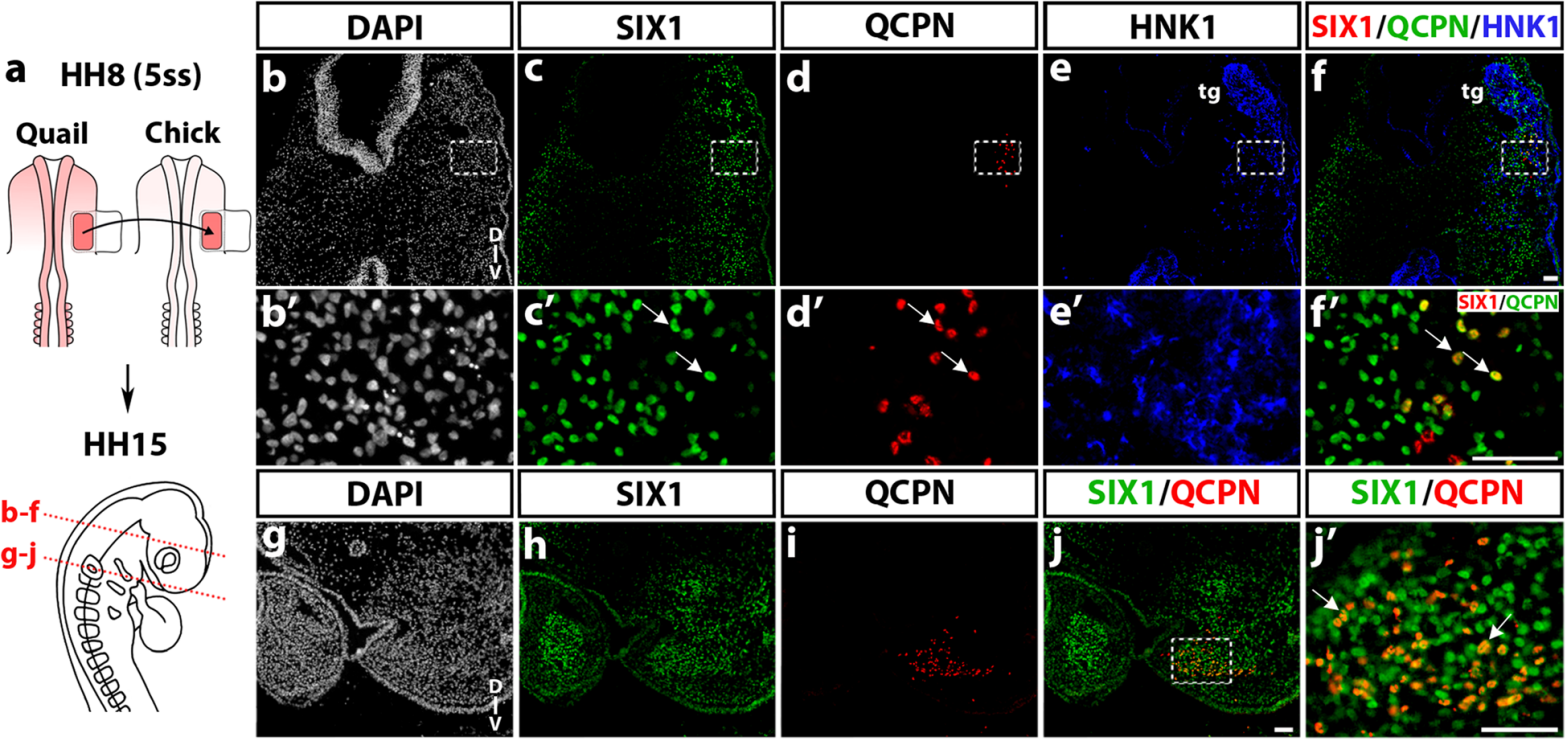
SIX1 expression in quail-chick chimeras of the cephalic mesoderm. **a** Schematic representation of the grafting procedure. Part of the chick lateral paraxial mesoderm was unilaterally replaced by its quail counterpart (in red) at stage HH8, and the chimeras were analyzed at HH15 (red dotted lines: position and orientation of the sections shown in **b**-**f** and **g**-**j**). **b**-**f** and **b’**-**f’**(magnfications of boxed areas) At the level of the trigeminal ganglion (tg) and nerves, detected by HNK1 staining (**e**, **f** and **e’**), engrafted quail mesodermal cells, co-stained with SIX1 and QCPN, are found in the host lateral mesoderm near the trigeminal ganglion (arrows in **c’**, **e’** and **f’**). **g**-**j** In the region of BA2, quail cells from the grafted mesoderm are present in the core mesenchyme of the BA, which strongly expresses SIX1. **j’** Magnification of the boxed area indicated in **j**, showing grafted mesodermal cells that express both SIX1 and QCPN (arrows). D-V, dorso-ventral orientation. Scale bars, 50 μm

The cranial mesoderm, located lateral to the mes-metencephalon, is known to yield various mesenchymal derivatives, including part of the chondrocranium and otic capsule, mandibular and dorsolateral extraocular muscles, meninges and blood vessel endothelium, which have been precisely mapped by quail-chick transplantations [2, 4, 36]. By performing the same type of cranial paraxial mesoderm transplantation, from quail to chicken embryos at stage HH8 (5ss) (Fig. 4a), we have explored the extent of SIX1 expression in the grafted mesodermal cells that developed in the chick host (stage HH15) 24–30 h after the transplantation. In cryosections at the level of the mesencephalon (Fig. 4b-f), SIX1-immunoreactive cell nuclei were widely distributed in the region of the forming trigeminal sensory ganglia and nerves, detected by expression of the NC marker HNK1 (Fig. 4e, *e’*). Ventral to the ganglion, a subset of engrafted quail mesodermal cells (QCPN+; Fig. 4d, *d’*) coexpressed SIX1 (Fig. 4 *f’*). More caudally, SIX1-positive cells occupied the core of BA2, where myogenic precursors will differentiate (Fig. 4h); on the same section, the engrafted quail mesodermal cells were located in a similar central position in the arch, and most of them exhibited SIX1-labeled nuclei (Fig. 4j, *j’*).

From these quail-chick grafting experiments we can conclude that, in the BAs, the central location of SIX1+ mesodermal cells is complementary to the peripheral distribution displayed by SIX1-negative NCC (see Fig. 3m). In more rostral head mesenchyme, deployment of SIX1 expression targeted mesenchymal cells derived from the mesoderm, such as in the vicinity of the trigeminal ganglion and from the NC, e.g., in the nasal and periocular regions.

### SIX1 expression in cephalic neuro-sensory structures

**Fig. 5:**
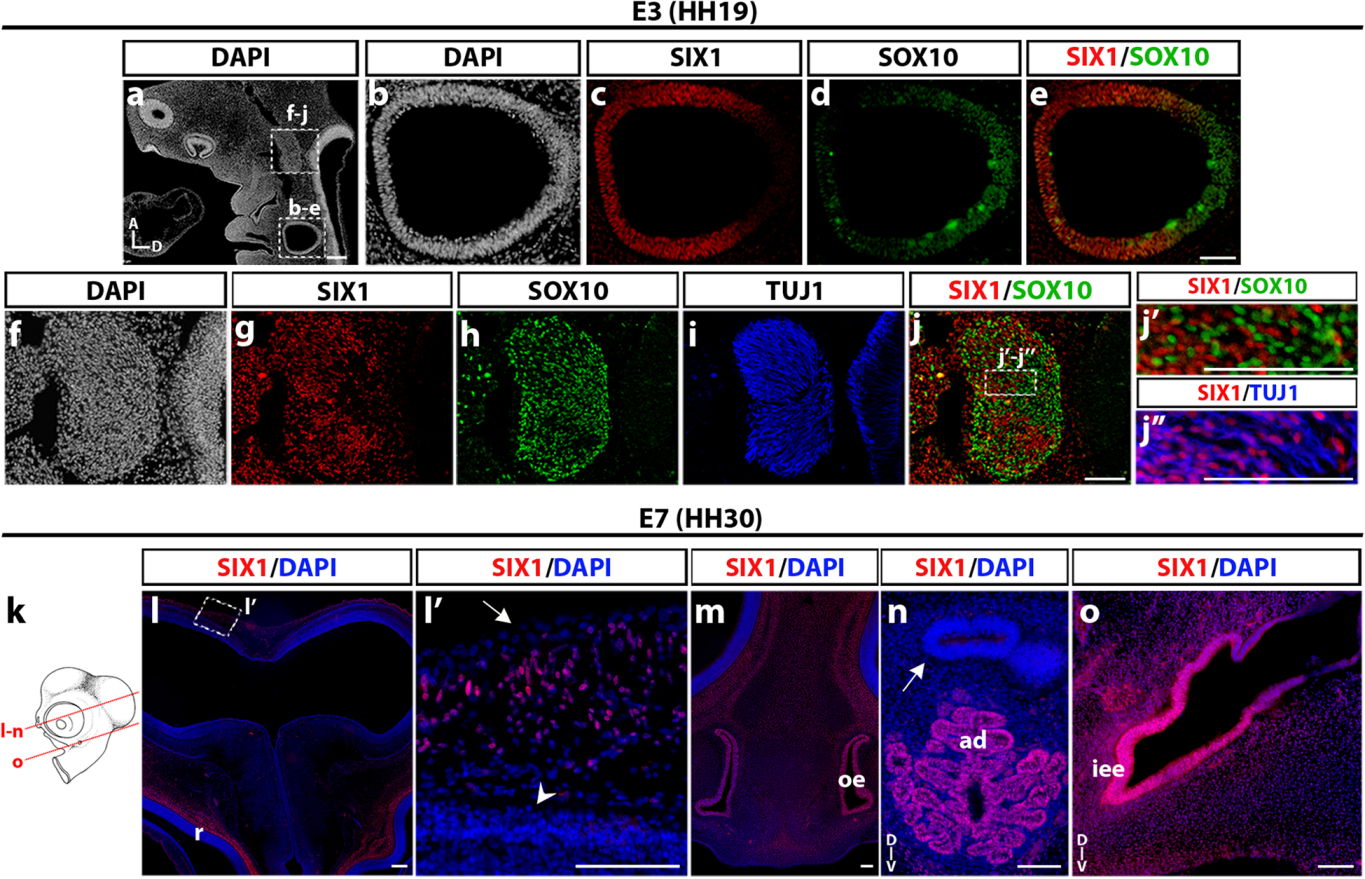
SIX1 expression in head neural and sensory structures of E3 and E7 chicken embryos. **a** DAPI staining of E3 (HH19) chicken head longitudinal section (A and D, anterior and dorsal orientations). Boxed areas indicate regions of the otic vesicle and trigeminal ganglion magnified in **b**-**e** and **f**-**j**, respectively. **c**-**e** SIX1 immunoreactivity is located in the ventrolateral region of the otic vesicle (**c**), whereas SOX10 labels its dorsal region (**d** and **e**). **f**-**j** More rostrally in the same section, SIX1-immunoreactive nuclei (**g**) exhibit a dense pattern in the trigeminal ganglion and surrounding mesenchyme; the trigeminal ganglion is positive for SOX10 (**h**) and TUJ1 (**i**) markers; **j** Merge of SIX1 and SOX10; **j’**-**j”** (magnifications of the boxed area indicated in **j**) SIX1-positive nuclei mostly correspond to SOX10-negative, TUJ1-positive neuronal cells in the trigeminal ganglion. **k** Schematic representation of a chick embryonic head at E7 (HH30); red lines indicate position and orientation of the sections shown in **n**-**o**. (**l**) SIX1-expressing cells are found lining the dorsal mesencephalon and more ventrally, adjacent to the retina (r). **l’** highlights the dorsal region boxed in **l,** showing SIX1-positive cells scattered in the mesenchyme between the surface ectoderm (arrow) and the neuroepithelium (arrowhead). **m** and **n** More ventrally in the same section, SIX1 is expressed in the olfactory epithelium (oe, in **m**), and adenohypophysis (ad, in **n**) whereas the neurohypophysis is SIX1-negative (arrow in **n**), and in the inner ear epithelium (iee in **o**). (D and V, dorsal and ventral orientations). Scale bars, 100 μm, except for **a** and **l**, 200 μm

We further studied the distribution of SIX1-expressing cells in the developing neural and sensory structures of the head of chicken embryos at E3 and E7. Fig. 5(a-j) shows a longitudinal section of an E3 (stage HH19) chicken head, where the otic vesicle exhibited strong SIX1 immunoreactivity (Fig. 5b,c), except in its dorsal part close to the rhombencephalon, which was labeled with SOX10 antibody (Fig. 5d and e), in agreement with the previously reported activity of a *SOX10* enhancer in early otic development [49]. In the mesencephalic region, SIX1 was abundantly expressed in the trigeminal ganglion (Fig. 5f, g), which was positive for SOX10 and TUJ1, markers of the non-neuronal and neuronal cells of the ganglion, respectively (Fig. 5h and i). In the core of the trigeminal ganglion, we did not observe a colocalization of SIX1 and SOX10 labeling in cell nuclei (Fig. 5j, *j’*), while SIX1 appeared to be expressed in the sensory neurons (Fig. 5 *j”*), in agreement with previous data [17, 44, 47].

In the E7 chicken head (Fig. 5k-o), SIX1 was detected in placodal derivatives, such as the olfactory epithelium (Fig. 5m), the adenohypophysis (Fig. 5n) and the epithelium of the inner ear (Fig. 5o). These results are consistent with the well-documented role of *SIX1* gene in the induction and maintenance of the ectodermal placodes, including its requirement for the formation of the middle and inner ear [13, 17, 50, 51]. In addition, in the region dorsal to the mesencephalon, dispersed SIX1-positive cell nuclei were identified in the mesenchyme between the neuroepithelium and the epidermis (Fig. 5l, *l’*), where meninges and skull bones later develop.

### SIX1 expression in differentiating cephalic mesenchymal tissues

**Fig. 6:**
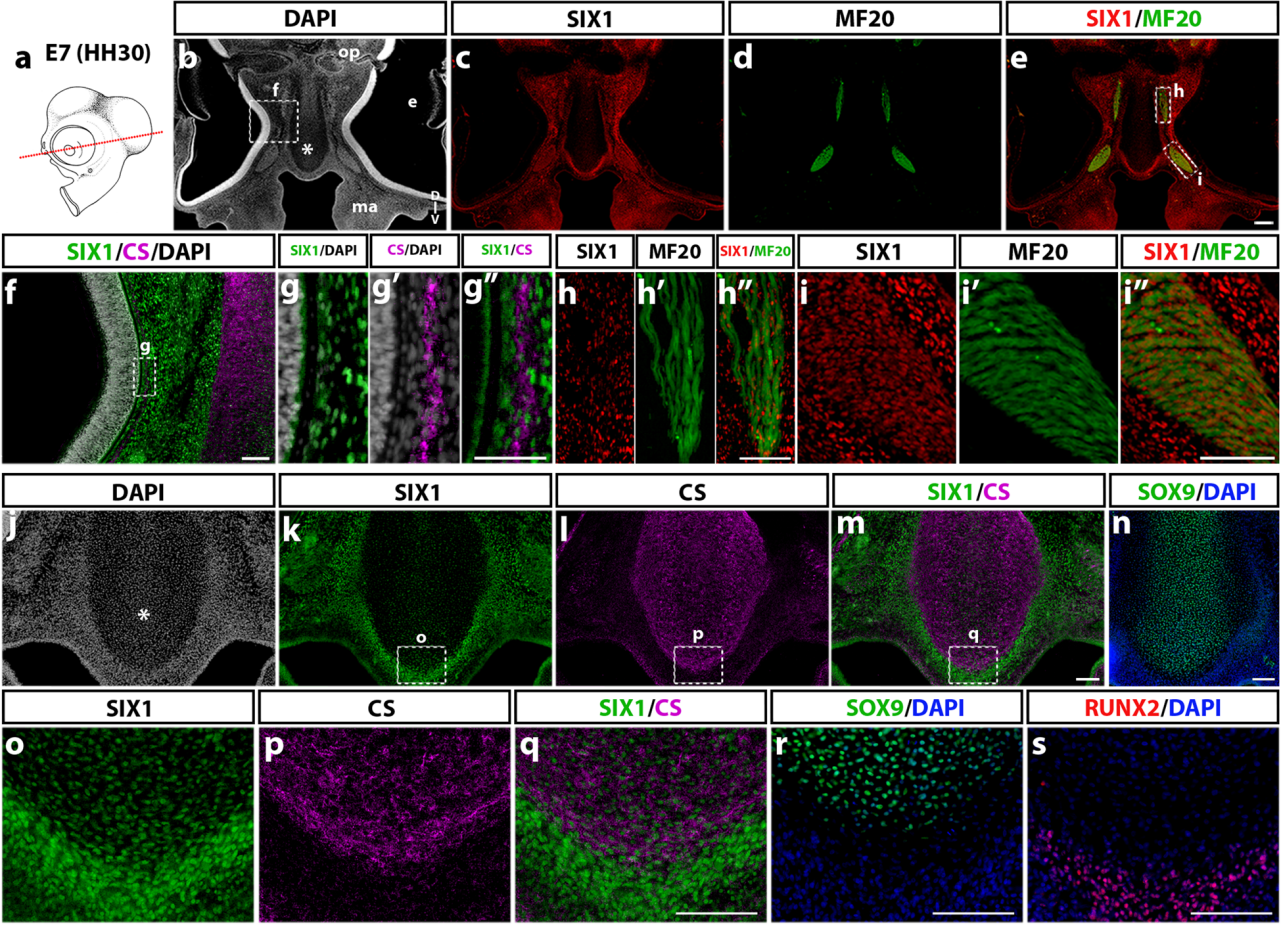
SIX1 expression in the nasal and periocular regions in E7 chicken. **a** Schematic representation of an E7 (HH30) chick embryo; the red line indicates the position and orientation of the section in **b**-**m** and **o**-**q**; adjacent sections are shown in **n**, **r** and **s**. (**b**-**e**) At the level of the eyes (e in **b**), SIX1 immunoreactivity (**c**) is widely detected in the periocular mesenchyme, nasal septum (* in **b**), maxillary bud (ma), and in extraocular muscles, positive for anti-myosin MF20 (**d**, **e**). **f** (Magnification of the area boxed in **b**) SIX1 expression pattern is dense in the periocular mesenchyme adjacent to the nasal septum positive for the chondrocytic marker CS. **g**-**g”** Highlights of the region boxed in **f**, showing SIX1 expression in the CS-positive scleral cartilage. **h** and **i** Magnifications of the areas boxed in **e**, showing SIX1 expression in medial rectus (**h**-**h”**) and inferior oblique (**i**-**i”**) extraocular muscles marked by MF20. **j-n** SIX1-positive nuclei are densely packed in the nasal mesenchyme and perichondrium while present at a lower density in the nasal septum (* in **j**), which is immunoreactive to CS (**l**, **m**) and SOX9 (**n**). **o**, **p**, **q** (magnifications of areas boxed in **k**, **l**, **m**) SIX1 is expressed in the CS-expressing nasal cartilage and, more strongly in its perichondrium; the latter tissues express the transcription factors SOX9 (**r**) and RUNX2 (**s**), respectively. D-V, dorso-ventral orientation; optic nerve (op). Scale bars, 100 μm, except for **b**-**e**, 300 μm and **g** *-* **g”**, 50 μm

We then focused on differentiated cephalic muscles and facial skeletal elements in the E7 chicken embryo and we examined the distribution of SIX1-positive cells in these tissues. Coronal sections at the ocular level (Fig. 6a) presented numerous cell nuclei labeled with SIX1 antibody in the nasal, maxillary and periocular mesenchyme (Fig. 6b, c), and in the region of the extraocular muscles (labeled with MF20 antibody; Fig. 6d, e). Detailed views of the same section showed that SIX1 is expressed in dispersed cells, close to the outer eye surface (Fig. 6f, g), in the region wherein the scleral cartilage is forming (Fig. 6 *g’*, *g”*) as well as in numerous cells within the extraocular muscles (medial rectus and inferior oblique muscles, Fig. 6h-*h”*, and i-*i*",- respectively). In the nasal septum (Fig. 6j-n), SIX1-immunoreactive cells were sparse in the area of chondrocytes labeled with CS and SOX9 (Fig. 6k-n and o-r) while highly enriched in the surrounding perichondrium (Fig. 6o), in which nuclei positive for the bone cell lineage marker RUNX2 were also detected (Fig. 6s).

**Fig. 7:**
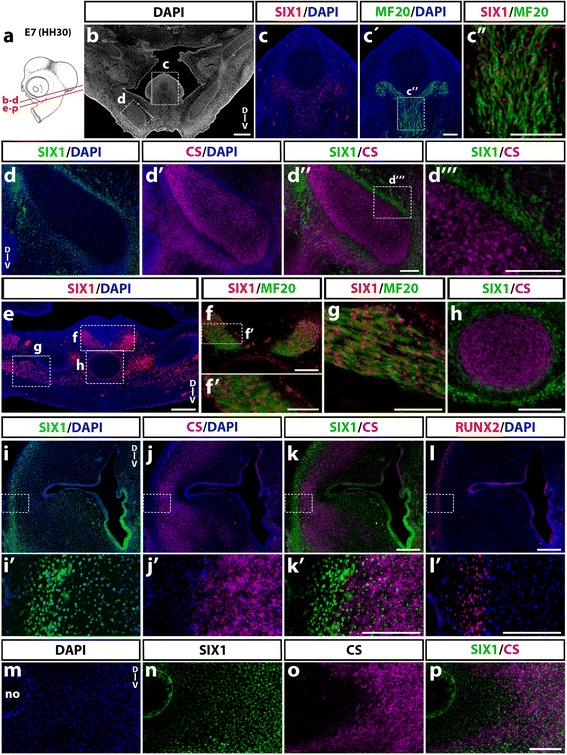
SIX1 expression in posterior cephalic muscles and skeletogenic mesenchyme in E7 chicken. **a** Schematic representation of an E7 (HH30) chick head; red line indicate the position and orientation of sections. **b**-**d** At BA1 level (**b**), SIX1 and MF20 (**c**-c') are co-expressed in tongue muscles (magnified in **c”**). **d”** In lower jaw rudiment, anti-CS labels Meckel’s cartilage, whereas SIX1 antibody only marks the perichondrium (see higher magnification in **d”’**). **e**-**h** SIX1, MF20 and CS staining in BA2 (**f**, **g**, **h**, magnifications of regions boxed in **e**). **e**-**g** SIX1 is expressed in laryngeal muscles, including ceratoglossus (**g**) and constrictor and dilatator glottidis (**f**-**f’**). **h** CS-labeled ceratobranchial cartilage, showing SIX1 expression only in the perichondrium. **i**-**l (i’**-**l’** magnifications of boxed areas) In the otocyst region, SIX1 immunostaining is present in the inner ear epithelium and adjacent CS-immunoreactive cartilage of the otic capsule (**i**-**k** and **i’**-**k’**) as well as in a more lateral area wherein cells bearing RUNX2 osteoblast marker were recorded (**l**-**l’**, adjacent section). **m**-**p** Partial overlap of SIX1 and CS markers in the ventral otic capsule adjacent to the notochord (no in **m**). D-V, dorso-ventral orientation. Scale bars, 100 μm, except for **b**, 300 μm; **f**, 50 μm; **e** and **i**-**l**, 200 μm

In more caudal sections (Fig. 7a), at the level of the jaw (Fig. 7b-*d*'''), SIX1 expression was found in tongue muscles (Fig. 7c-*c”*) and in cells lining Meckel’s cartilage in the lower jaw while the CS-synthesizing cartilage itself was negative (Fig. 7d-*d*'''). In sections at the level of the throat (Fig. 7e-g), laryngeal muscles (Fig. 7e-g) and the periphery of the ceratobranchial cartilage (Fig. 7e, h), were positive for SIX1. In the ear region (Fig. 7i-p), SIX1 was widely expressed in the otic mesenchyme, in addition to the previously mentioned otic epithelium (Fig. 7i, *i’*). The distal part of the otic capsule comprised SIX1-positive cell nuclei both within the area of chondrocytes (immunoreactive to CS; (Fig. 7j, *j’* and k, *k’*) and at its periphery, in the region of presumptive cranial bones, wherein cells immunoreactive to RUNX2 were recorded (Fig. 7l, *l’*). SIX1 was also widely expressed in the ventral part of the otic capsule (Fig. 7m, n), including the region of chondrocytes that produced CS (Fig. 7o, p).

These data show that SIX1 expression in the E7 head skeletogenic mesenchyme displays a complex pattern: while present in some cartilages (e.g., lateral and ventral otic capsule), in others such as Meckel’s and ceratobranchial cartilages, it is detected only in the perichondrium. In addition, head muscles (including extraocular, pharyngeal, tongue and laryngeal muscles) exhibit strong expression of SIX1, indicating that this factor could be a crucial regulator of cranial myogenesis in the avian embryo, as described in zebrafish [52] and in the limb and trunk skeletal muscles of the chick and mouse [15, 18, 53, 54]*.*

### Differentiation of SIX1-expressing cells in cranial NCC cultures

**Fig. 8:**
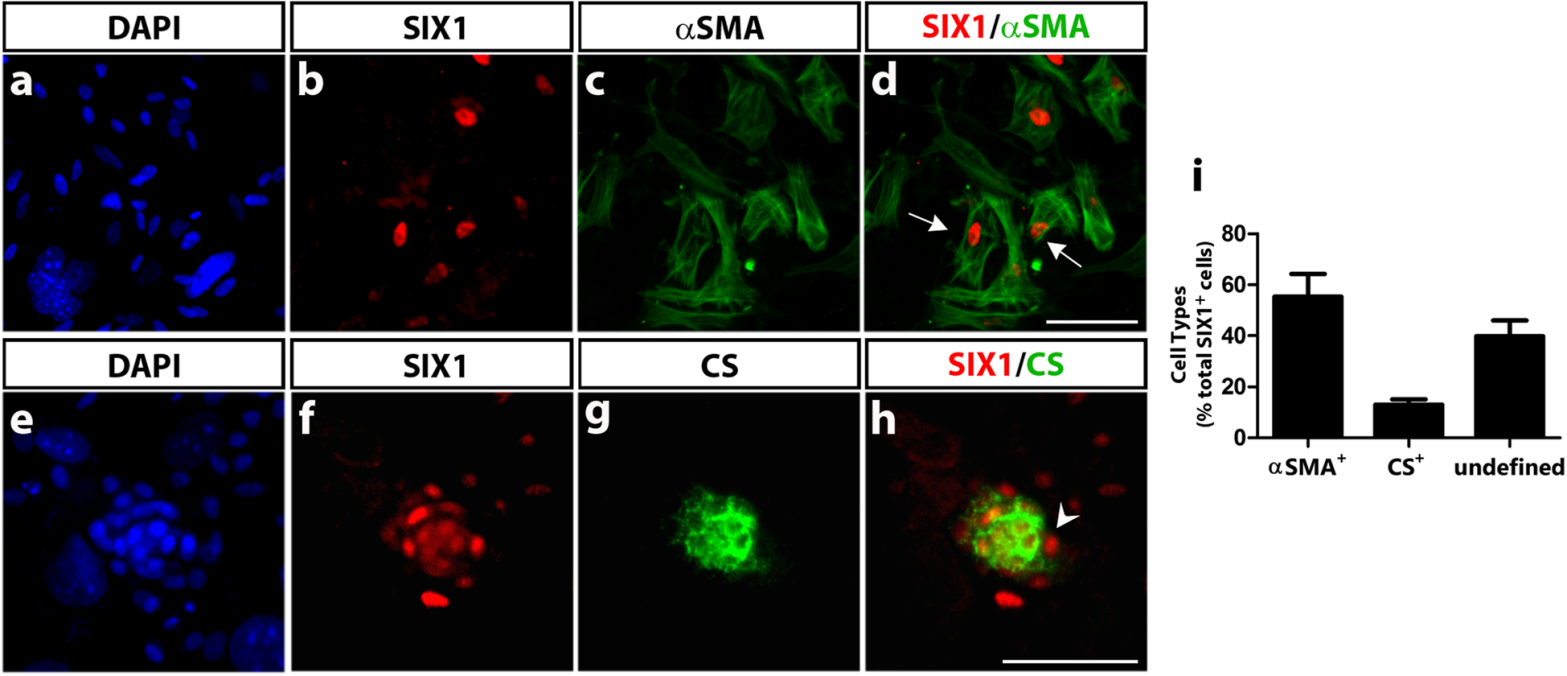
SIX1 expression in cephalic NCC differentiating in culture. After 6 days of culture, cephalic quail NCC were assayed for expression of SIX1 and various NC-derived phenotypic markers (see Methods section). SIX1-positive nuclei were recorded in approximately 1% of the NCC population (*n* = 12 cultures). **a-d** Co-staining of nuclear SIX1 and cytoplasmic αSMA shows expression of SIX1 in myofibroblastic cells (arrows in **d**). **e**-**h** DAPI, SIX1 and CS co-staining identifies SIX1-expressing chondrocytes, positive for CS and forming a tridimensional nodule; SIX1-immunoreactive cells are also present in the perichondrial region (arrowhead in **h**). **i** Quantification of cell phenotypes in the SIX1-positive cell subpopulation identified in the NCC cultures. Data are expressed as mean ± S.E.M. (*n* = 5 cultures). Approximately 55% of SIX1-labeled NCC were of myofibroblastic (αSMA+) and 13% of chondrocytic (CS+), phenotypes. The remaining SIX1-expressing cells (i.e., negative for CS and αSMA) were not labeled with HNK1 and tyrosine hydroxylase (markers of PNS precursor cells and adrenergic neurons, respectively); these cells are thus designated as of undefined phenotype. Scale bars, 50 μm

To gain further insights on the pattern of expression of SIX1 in the cephalic NCC, we have investigated whether the SIX1 protein is present in the distinct cell types that these NCC can produce, when cultured in vitro in conditions previously shown to be appropriate for the development of multipotent NC progenitors [38, 39, 55]. Briefly, early cephalic NCC were obtained after their emigration from mes-metencephalon explanted from quail embryos at 6-7ss and cultured for 18 h; isolated NCC were thereafter subcultured and maintained for 6 days in vitro, as we previously reported [38]. Phenotype analysis with cell type-specific antibodies indicated the differentiation of neurons and glial cells, melanoblasts, myofibroblasts and chondrocytes, as expected. Simultaneous detection of SIX1-immunoreactivity in the cephalic NCC cultures revealed that two differentiated NC-derived cell types expressed SIX1: myofibroblasts positive for the smooth muscle marker αSMA (Fig. 8a-d) and chondrocytes, within cartilage nodules that synthesized CS (Fig. 8e-h). The other, neural and melanocytic, cells derived from the NCC, were negative for SIX1 (not shown). In addition to labeling mesenchymal cells (myofibroblasts and chondrocytes), SIX1 was also expressed in the nuclei of a subset of NCC with an undefined phenotype (since these cells were also negative for markers of the neuronal, glial and melanocytic lineages). Cell quantification indicated that the majority of SIX1-positive cells were myofibroblasts (Fig. 8i). In fact, SIX1-expressing cells represented a very small subpopulation of the NCC (approximately 1%) in these 6 day-cultures, and were not detected at earlier culture time-points (not shown). Therefore, at least in these in vitro conditions, expression of SIX1 was acquired during the differentiation of myofibroblasts and chondrocytes. The in vitro expression of SIX1 in αSMA+ cells is rather puzzling since we did not observe SIX1-immunoreactivity in the NC-derived pericytes and smooth muscle cells lining vascular endothelia in E7 chicken head (Additional file 2: Figure S2). However, αSMA is rather a versatile marker in vitro, labeling a population of fibroblastic-like cells that develop in cultures of avian and mouse NCC, particularly under stimulation by TGFβ [56]. Further analyses are required to assign a definitive identity, of connective or smooth muscle cells, to this particular subset of SIX1-positive cells differentiating in cephalic NCC cultures.

## Discussion

The development of the head of vertebrates relies upon a series of morphogenetic movements, which trigger coordinated interactions of cell populations of distinct embryonic origin. It is particularly evidenced in the mesenchymal tissues of the head, which are composed of cells from a dual embryonic source: mesoderm and NC. Some examples of the importance of coordinated communication between mesenchymal tissues are: the definition of mesoderm/NC boundary in cranial bone sutures, particularly the coronal suture of mammals [57–59]; the recruitment of NC-derived pericytes and vascular smooth muscle cells to the endothelium in the forming blood vessels (of mesoderm origin) [5]; the coordination between mesoderm and NC-derived cartilage precursors for the correct development of otic capsule, and the patterning of head muscles, due to interactions between mesodermal myogenic cells and connective cells/tenocytes of NC origin [36, 46, 60].

Here we have considered the respective contribution of cranial NC and mesoderm to the territories that express *SIX1* gene and SIX1 protein in the developing avian head. We first observed SIX1 expression in many ectodermal derivatives, such as the olfactory, otic and epibranchial placodes. This corroborates current literature, which classifies *SIX1* gene as a pan-placodal marker [12, 13].

SIX1 expression in head mesenchymal tissues has been reported in mouse and chicken embryos [17, 33, 44]. Here, we have studied the distribution of SIX1-expressing cells in the avian embryo between stage HH11 and E7, focusing on their origin and differentiation fate.

In early developmental stages (HH11), during NCC migration, we found that SIX1 is mainly present in the cranial mesenchyme of non-NC origin. This was attested by the lack of colocalization with SOX10, a NC specifier factor expressed in the NCC at migratory stages. This result contradicts a previous report of *SIX1* mRNA expression in premigratory and migratory cranial NCC in whole-mount preparations of the chick embryo [33]. However, a recent detailed study of gene expression during formation of the neural fold has shown that only a subtle set of early cranial NCC express SIX1 transiently at this stage, when SIX1 expression is mainly observed in the future placodal region [61].

At late migratory NC stages, in E2.5 chicken, SIX1 had a broad expression in the head mesenchyme, especially in the periocular mass and surrounding the developing forebrain. By fate analyses performed in quail-chick chimeras, we could definitely distinguish between NC and mesoderm mesenchymal populations. After quail neural fold transplantation into the chick embryo, SIX1-expressing NCC populated the periocular, perinasal and periotic mesenchyme. Following the transplantation of quail cranial paraxial mesoderm into the chick embryo, a subset of mesodermal donor cells displayed SIX1 expression in the preotic mesenchyme, closely located to the developing trigeminal ganglion. Therefore, cells originating from either the NC or mesoderm switch on SIX1 at these early stages of development, hence SIX1 does not represent a reliable marker of the origin of cranial mesenchymal cells. Intriguingly, in the BAs, SIX1 was mostly expressed in the core of the mesenchyme, known to be mesoderm-derived [2, 36, 60] (see also Fig. 4), which suggests that SIX1 is an early marker of jaw muscles. Our grafting experiments clearly showed that SIX1-expressing cells in the center of BAs, are derived from the mesoderm and distinct from the NC-derived cells in the arch periphery. It is rather puzzling that we did not identify SIX1-expressing cells in the pharyngeal NC-mesenchyme since *SIX1* null mice exhibit some craniofacial anomalies related with NC-derived skeletal elements of the BAs. Whether this could be due to an indirect effect of *SIX1* loss in the presumptive muscles or from loss of *SIX1* expression in these skeletal tissues at later stages remains to be investigated. Nevertheless, our data show that SIX1 expression in NC-derived tissues, at these early stages, appears to be restricted to the preotic mesenchyme.

We also described SIX1 expression at later stages, in E3 and E7 chicken. At E3, SIX1 was expressed in the ventral otic placode, where it is essential for cochlear and vestibular development [62]. Moreover, SIX1 was present in neurons of the trigeminal ganglion, but not in SOX10+ glial progenitors. Similar results have been described in mouse embryos [47].

Regarding mesenchymal cell types, at E7, SIX1 was present in the nasal and periocular mesenchyme and in cartilage components of the developing eye, such as the scleral cartilage, which are of NC origin [63]. Of note, no expression of SIX1 was detected in eye structures such as the retina, lens, cornea and optic nerve. These results corroborate previous findings in the mouse showing that *SIX1*, and the closely-related gene *SIX2*, are not involved in ocular development of vertebrates, although they have an evolutionary homology with *Sine oculis* of *Drosophila* [6]. Interestingly, in the NC-derived nasal septum [2], SIX1 was expressed both in chondrocytes and perichondrium, although its expression was much stronger in the latter. SIX1-positive chondrocytes were also recorded in several regions of the otic capsule at E7 while other cartilages showed expression of SIX1 restricted to perichondrial cells, (i.e., Meckel and ceratobranchial).

Furthermore, SIX1 expression by the chondrocytes derived from NCC was found in cultures of cephalic NCC, albeit the total number of SIX1-positive cells in these cultures was rather limited (about 1%). Finally, we observed SIX1-expressing cells dispersed in the mesenchyme adjacent to the dorsal surface of the mesencephalon, which suggests SIX1 expression in precursors of the meninges, which are mesodermal-derived in this brain region [36].

Regarding mesodermal derivatives, we have also shown that SIX1 is mainly expressed in head muscles, including the extraocular muscles, the branchiomeric muscles of the pharyngeal and laryngeal regions, and tongue muscles. Indeed, in *SIX1* null mice, these muscles are greatly impaired, together with an extensive hypoplasia of the trunk and limb muscles, which require *SIX1* function at several steps of myogenesis [15, 17]. These findings suggest a common dependence on *SIX1* gene for a general skeletal muscle developmental program. The exact role of *SIX1* gene in head muscles should be further investigated, since genetic networks regulating patterning and differentiation are distinct between developing head and trunk muscles [64]. Finally, at least in the head, SIX1 appeared to be deprived of any contribution to the vascular system: we did not observe SIX1 expression in head blood vessels at all stages analyzed neither in NC-derived vascular smooth muscle cells and pericytes at E7.

In summary, the careful examination of SIX1 protein expression in mesenchymal tissues of the developing avian embryo from stage HH11 to E7 and in quail-chick chimeras shows that this gene is expressed in cells derived from both cranial NC and mesoderm, in a tissue- and stage- dependent manner. These results open the way to investigate *SIX1* downstream targets and further decipher *SIX1* gene function in the establishment of the diverse mesenchymal cranial tissues. Future experiments in which one could selectively knockdown *SIX1*, in a temporal and tissue-specific way, would be beneficial for a further understanding of its role in mesoderm and NC patterning and differentiation during head development.

## Conclusions

During vertebrate head development, mesenchymal tissues (e.g., bones, cartilages, dermis, muscles) differentiate and assemble from initially two main populations of cranial progenitor cells, which originate from either the NC or the mesoderm. The molecular players that orchestrate co-development of cephalic NCC and mesodermal cells to properly construct the head remain poorly understood. We present here the dynamics of the expression of SIX1 protein, during migration of cephalic progenitor cells and their development into head mesenchymal tissues in the avian embryo. Our data reveal that this gene is expressed in cells derived from both cranial NC and mesoderm, in a tissue- and stage-dependent manner. This will be useful to investigate how *SIX1* gene can act in the coordinated development of these two cellular populations during vertebrate head morphogenesis.

## Additional files

Additional file 1: Figure S1. SIX1 expression in control quail embryos**. a** Schematic of a quail embryo at HH15 (red dotted lines indicate the position and orientation of the sections shown in **b**, **c** and **d**, **e**). Sections are labeled with the quail-specific marker QCPN (**b**, **d**); SIX1 expression in found in the periocular mesenchyme (**c**), and in the ectoderm, endoderm as well as the core of the BA (**e**). This expression pattern is similar to that described at the same stage in control chick embryos (see Fig. 2 **i**, **j**) and in quail-chick chimeras (see Fig. 3 **g**, **k** and Fig. 4 **h**). Scale bars, 100 μm. (TIFF 601 kb)

Additional file 2: Figure S2. Absence of SIX1-immunoreactive cells in head blood vessels at E5. (**a, d, g**) SIX1 immunostaining in sections of an E5 (HH25) chicken head, counterstained with DAPI. (**b, e, h**) αSMA immunostaining and DAPI. (**c, f, i**) Merge of SIX1 and αSMA labeling. (**d-f**) Magnifications of the area depicted in **a-c**; note SIX1 expression in head mesenchyme (hm in **d**) but not in the wall of blood vessels (arrowhead in **f**) adjacent to the neuroepithelium (np in **d**), neither in αSMA+ pericytes within the brain epithelium (arrows in **f**). **g-i** Magnifications of the area depicted in **a-c**; observe strong SIX1 staining (**g**) in the inner ear epithelium and associated vestibulo-acoustic ganglion (gVIII), whereas αSMA+ smooth muscle cells lining blood vessels lack SIX1 expression (arrow in **i**). Scale bars, 100 μm, except in **a-c**, 300 μm. (TIFF 3552 kb)

## Abbreviations

BA: Branchial arch
HH: Hamburger and Hamilton
NC: Neural crest
NCC: Neural crest cells
PNS: Peripheral nervous system
Ss: Somite-stage

## References

[1] Gans C, Northcutt RG (1983). Neural Crest and the Origin of Vertebrates: A New Head. Science.

[2] Couly GF, Coltey PM, Le Douarin NM (1993). The triple origin of skull in higher vertebrates: a study in quail-chick chimeras. Development.

[3] Creuzet S, Couly G, Le Douarin NM (2005). Patterning the neural crest derivatives during development of the vertebrate head: insights from avian studies. J Anat.

[4] Couly G, Coltey P, Eichmann A, Le Douarin NM (1995). The angiogenic potentials of the cephalic mesoderm and the origin of brain and head blood vessels. Mech Dev.

[5] Etchevers HC, Vincent C, Le Douarin NM, Couly GF (2001). The cephalic neural crest provides pericytes and smooth muscle cells to all blood vessels of the face and forebrain. Development.

[6] Kawakami K, Sato S, Ozaki H, Ikeda K (2000). Six family genes-structure and function as transcription factors and their roles in development. BioEssays.

[7] Kumar JP (2009). The sine oculis homeobox (SIX) family of transcription factors as regulators of development and disease. Cell Mol Life Sci.

[8] Lagutin OV, Zhu CC, Kobayashi D, Topczewski J, Shimamura K, Puelles L (2003). Six3 repression of Wnt signaling in the anterior neuroectoderm is essential for vertebrate forebrain development. Genes Dev.

[9] Oliver G, Mailhos A, Wehr R, Copeland N, Jenkins N, Gruss P (1995). Six3, a murine homologue of the sine oculis gene, demarcates the most anterior border of the developing neural plate and is expressed during eye development. Development.

[10] Lavado A, Lagutin OV, Oliver G (2008). Six3 inactivation causes progressive caudalization and aberrant patterning of the mammalian diencephalon. Development.

[11] Jeong Y, Leskow FC, El-Jaick K, Roessler E, Muenke M, Yocum A (2008). Regulation of a remote Shh forebrain enhancer by the Six3 homeoprotein. Nat Genet.

[12] Baker CV, Bronner-Fraser M (2001). Vertebrate cranial placodes I. Embryonic induction. Dev. Biol..

[13] Schlosser G (2010). Making Senses. Development of Vertebrate Cranial Placodes. Int. Rev. Cell Mol. Biol..

[14] Oliver G, Wehr R, Jenkins NA, Copeland NG, Cheyette BN, Hartenstein V (1995). Homeobox genes and connective tissue patterning. Development.

[15] Laclef C, Hamard G, Demignon J, Souil E, Houbron C, Maire P (2003). Altered myogenesis in Six1-deficient mice. Development.

[16] Bonnin MA, Laclef C, Blaise R, Eloy-Trinquet S, Relaix F, Maire P (2005). Six1 is not involved in limb tendon development, but is expressed in limb connective tissue under Shh regulation. Mech Dev.

[17] Laclef C, Souil E, Demignon J, Maire P (2003). Thymus, kidney and craniofacial abnormalities in Six1 deficient mice. Mech Dev.

[18] Heanue TA, Reshef R, Davis RJ, Mardon G, Oliver G, Tomarev S (1999). Synergistic regulation of vertebrate muscle development by Dach2, Eya2, and Six1, homologs of genes required for Drosophila eye formation. Genes Dev.

[19] Ruf RG, Xu P-X, Silvius D, Otto EA, Beekmann F, Muerb UT (2004). SIX1 mutations cause branchio-oto-renal syndrome by disruption of EYA1-SIX1-DNA complexes. Proc Natl Acad Sci U S A.

[20] Kochhar A, Orten DJ, Sorensen JL, Fischer SM, Cremers CWRJ, Kimberling WJ (2008). SIX1 mutation screening in 247 branchio-oto-renal syndrome families: A recurrent missense mutation associated with BOR. Hum Mutat.

[21] Guan J, Wang D, Cao W, Zhao Y, Du R, Yuan H (2016). SIX2 haploinsufficiency causes conductive hearing loss with ptosis in humans. J Hum Genet.

[22] Hufnagel RB, Zimmerman SL, Krueger LA, Bender PL, Ahmed ZM, Saal HM (2016). A new frontonasal dysplasia syndrome associated with deletion of the SIX2 gene. Am J Med Genet Part A.

[23] He G, Tavella S, Hanley KP, Self M, Oliver G, Grifone R (2010). Inactivation of Six2 in mouse identifies a novel genetic mechanism controlling development and growth of the cranial base. Dev Biol.

[24] Chai Y, Jiang X, Ito Y, Bringas P, Han J, Rowitch DH (2000). Fate of the mammalian cranial neural crest during tooth and mandibular morphogenesis. Development.

[25] Jiang X, Iseki S, Maxson RE, Sucov HM, Morriss-Kay GM (2002). Tissue Origins and Interactions in the Mammalian Skull Vault. Dev Biol.

[26] Santagati F, Rijli FM (2003). Cranial neural crest and the building of the vertebrate head. Nat Rev Neurosci.

[27] Kutejova E, Engist B, Mallo M, Kanzler B, Bobola N (2005). Hoxa2 downregulates Six2 in the neural crest-derived mesenchyme. Development.

[28] Kutejova E, Engist B, Self M, Oliver G, Kirilenko P, Bobola N (2008). Six2 functions redundantly immediately downstream of Hoxa2. Development.

[29] Couly G, Grapin-botton A, Coltey P, Ruhin B, Le Douarin NM. Determination of the identity of the derivatives of the cephalic neural crest: incompatibility between Hox gene expression and lower jaw development. Development 1998;3459:3445–3459. 10.1242/dev.125.17.3445 9693148

[30] Couly G, Creuzet S, Bennaceur S, Vincent C, Le Douarin NM (2002). Interactions between Hox-negative cephalic neural crest cells and the foregut endoderm in patterning the facial skeleton in the vertebrate head. Development.

[31] Creuzet S, Couly G, Vincent C, Le Douarin NM (2002). Negative effect of Hox gene expression on the development of the neural crest-derived facial skeleton. Development.

[32] Creuzet SE (2009). Neural crest contribution to forebrain development. Semin Cell Dev Biol.

[33] Garcez RC, Le Douarin NM, Creuzet SE (2014). Combinatorial activity of Six1-2-4 genes in cephalic neural crest cells controls craniofacial and brain development. Cell Mol Life Sci.

[34] Hamburger V, Hamilton H (1951). A series of normal stages in the development of the chick embryo. J Morphol.

[35] Couly GF, Le Douarin NM (1987). Mapping of the early neural primordium in quail-chick chimeras. II. The prosencephalic neural plate and neural folds: Implications for the genesis of cephalic human congenital abnormalities. Dev. Biol.

[36] Couly GF, Coltey PM, Le Douarin NM (1992). The developmental fate of the cephalic mesoderm in quail-chick chimeras. Development.

[37] Marillat V, Cases O, Nguyen-Ba-Charvet KT, Tessier-Lavigne M, Sotelo C, Chedotal A (2002). Spatiotemporal expression patterns of slit and robo genes in the rat brain. J Comp Neurol.

[38] Calloni GW, Glavieux-Pardanaud C, Le Douarin NM, Dupin E (2007). Sonic Hedgehog promotes the development of multipotent neural crest progenitors endowed with both mesenchymal and neural potentials. Proc Natl Acad Sci U S A.

[39] Calloni GW, Le Douarin NM, Dupin E (2009). High frequency of cephalic neural crest cells shows coexistence of neurogenic, melanogenic, and osteogenic differentiation capacities. Proc Natl Acad Sci U S A.

[40] Le Douarin NM, Kalcheim C (1999). The Neural Crest.

[41] Fauquet M, Ziller C (1989). A monoclonal antibody directed against quail tyrosine hydroxylase: description and use in immunocytochemical studies on differentiating neural crest cells. J Histochem Cytochem.

[42] Baroffio A, Dupin E, Le Douarin NM (1988). Clone-forming ability and differentiation potential of migratory neural crest cells. Proc Natl Acad Sci U S A.

[43] Mootoosamy RC, Dietrich S (2002). Distinct regulatory cascades for head and trunk myogenesis. Development.

[44] Sato S, Ikeda K, Shioi G, Nakao K, Yajima H, Kawakami K (2012). Regulation of Six1 expression by evolutionarily conserved enhancers in tetrapods. Dev Biol.

[45] Cheng Y, Cheung M, Abu-Elmagd MM, Orme A, Scotting PJ (2000). Chick sox10, a transcription factor expressed in both early neural crest cells and central nervous system. Brain Res Dev Brain Res.

[46] Noden DM, Trainor PA (2005). Relations and interactions between cranial mesoderm and neural crest populations. J Anat.

[47] Karpinski BA, Bryan CA, Paronett EM, Baker JL, Fernandez A, Horvath A (2016). A cellular and molecular mosaic establishes growth and differentiation states for cranial sensory neurons. Dev Biol.

[48] Couly G, Le Douarin NM (1988). The fate map of the cephalic neural primordium at the presomitic to the 3-somite stage in the avian embryo. Development.

[49] Betancur P, Bronner-Fraser M, Sauka-Spengler T (2010). Genomic code for Sox10 activation reveals a key regulatory enhancer for cranial neural crest. Proc Natl Acad Sci U S A.

[50] Zheng W, Huang L, Wei Z-B, Silvius D, Tang B, Xu P-X (2003). The role of Six1 in mammalian auditory system development. Development.

[51] Grocott T, Tambalo M, Streit A (2012). The peripheral sensory nervous system in the vertebrate head: a gene regulatory perspective. Dev Biol.

[52] Lin CY, Chen WT, Lee HC, Yang PH, Yang HJ, Tsai HJ (2009). The transcription factor Six1a plays an essential role in the craniofacial myogenesis of zebrafish. Dev Biol.

[53] Grifone R, Demignon J, Houbron C, Souil E, Niro C, Seller MJ (2005). Six1 and Six4 homeoproteins are required for Pax3 and Mrf expression during myogenesis in the mouse embryo. Development.

[54] Grifone R, Demignon J, Giordani J, Niro C, Souil E, Bertin F (2007). Eya1 and Eya2 proteins are required for hypaxial somitic myogenesis in the mouse embryo. Dev Biol.

[55] Coelho-Aguiar JM, Le Douarin NM, Dupin E (2013). Environmental factors unveil dormant developmental capacities in multipotent progenitors of the trunk neural crest. Dev Biol.

[56] Dupin E, Coelho-Aguiar JM (2013). Isolation and differentiation properties of neural crest stem cells. Cytometry A.

[57] Merrill AE, Bochukova EG, Brugger SM, Ishii M, Pilz DT, Wall SA (2006). Cell mixing at a neural crest-mesoderm boundary and deficient ephrin-Eph signaling in the pathogenesis of craniosynostosis. Hum Mol Genet.

[58] Ting M-C, Wu NL, Roybal PG, Sun J, Liu L, Yen Y (2009). EphA4 as an effector of Twist1 in the guidance of osteogenic precursor cells during calvarial bone growth and in craniosynostosis. Development.

[59] Deckelbaum RA, Holmes G, Zhao Z, Tong C, Basilico C, Loomis CA (2012). Regulation of cranial morphogenesis and cell fate at the neural crest-mesoderm boundary by engrailed 1. Development.

[60] Grenier J, Teillet MA, Grifone R, Kelly RG, Duprez D (2009). Relationship between neural crest cells and cranial mesoderm during head muscle development. PLoS One.

[61] Roellig D, Tan-Cabugao J, Esaian S, Bronner ME (2017). Dynamic transcriptional signature and cell fate analysis reveals plasticity of individual neural plate border cells. elife.

[62] Ozaki H, Nakamura K, Funahashi JI, Ikeda K, Yamada G, Tokano H (2004). Six1 controls patterning of the mouse otic vesicle. Development.

[63] Creuzet S, Vincent C, Couly G (2005). Neural crest derivatives in ocular and periocular structures. Int J Dev Biol.

[64] Sambasivan R, Kuratani S, Tajbakhsh S (2011). An eye on the head: the development and evolution of craniofacial muscles. Development.

