# Supplementary figures and images for: Respective contribution of the cephalic neural crest and mesoderm to SIX1-expressing head territories in the avian embryo
Source: BMC Dev Biol. 2017 Oct 10;17:13. doi: 10.1186/s12861-017-0155-z (PMC5634862; doi:10.1186/s12861-017-0155-z)

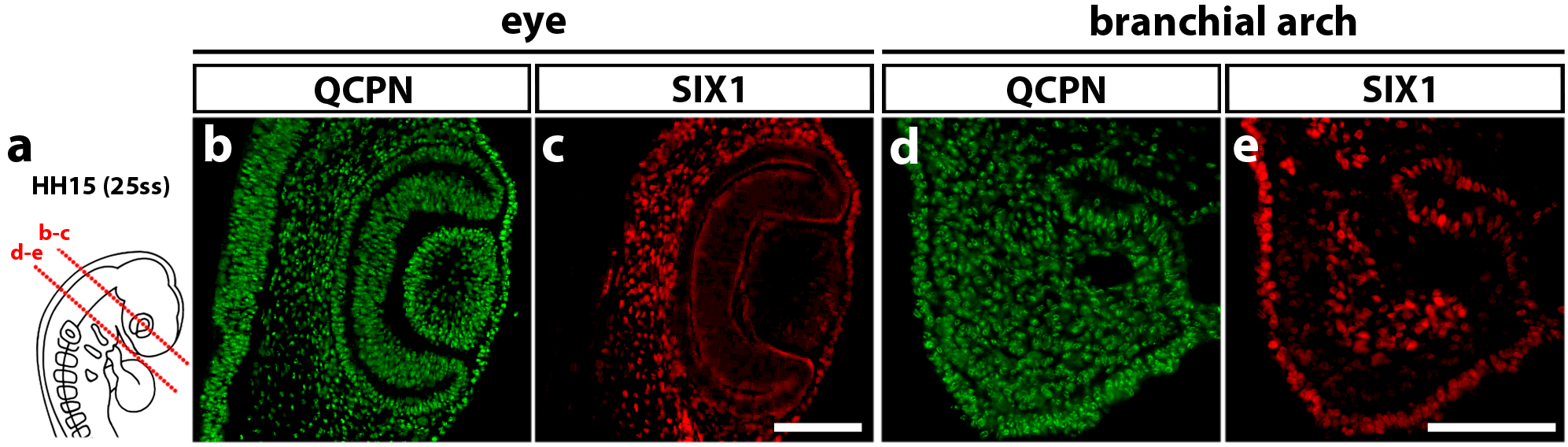

Supplement: Supplementary file 1 — SIX1 expression in control quail embryos. a Schematic of a quail embryo at HH15 (red dotted lines indicate the position and orientation of the sections shown in b, c and d, e). Sections are labeled with the quail-specific marker QCPN (b, d); SIX1 expression in found in the periocular mesenchyme (c), and in the ectoderm, endoderm as well as the core of the BA (e). This expression pattern is similar to that described at the same stage in control chick embryos (see Fig. 2 i, j) and in quail-chick chimeras (see Fig. 3 g, k and Fig. 4 h). Scale bars, 100 μm. (TIFF 601 kb) [file 12861_2017_155_MOESM1_ESM.tif]

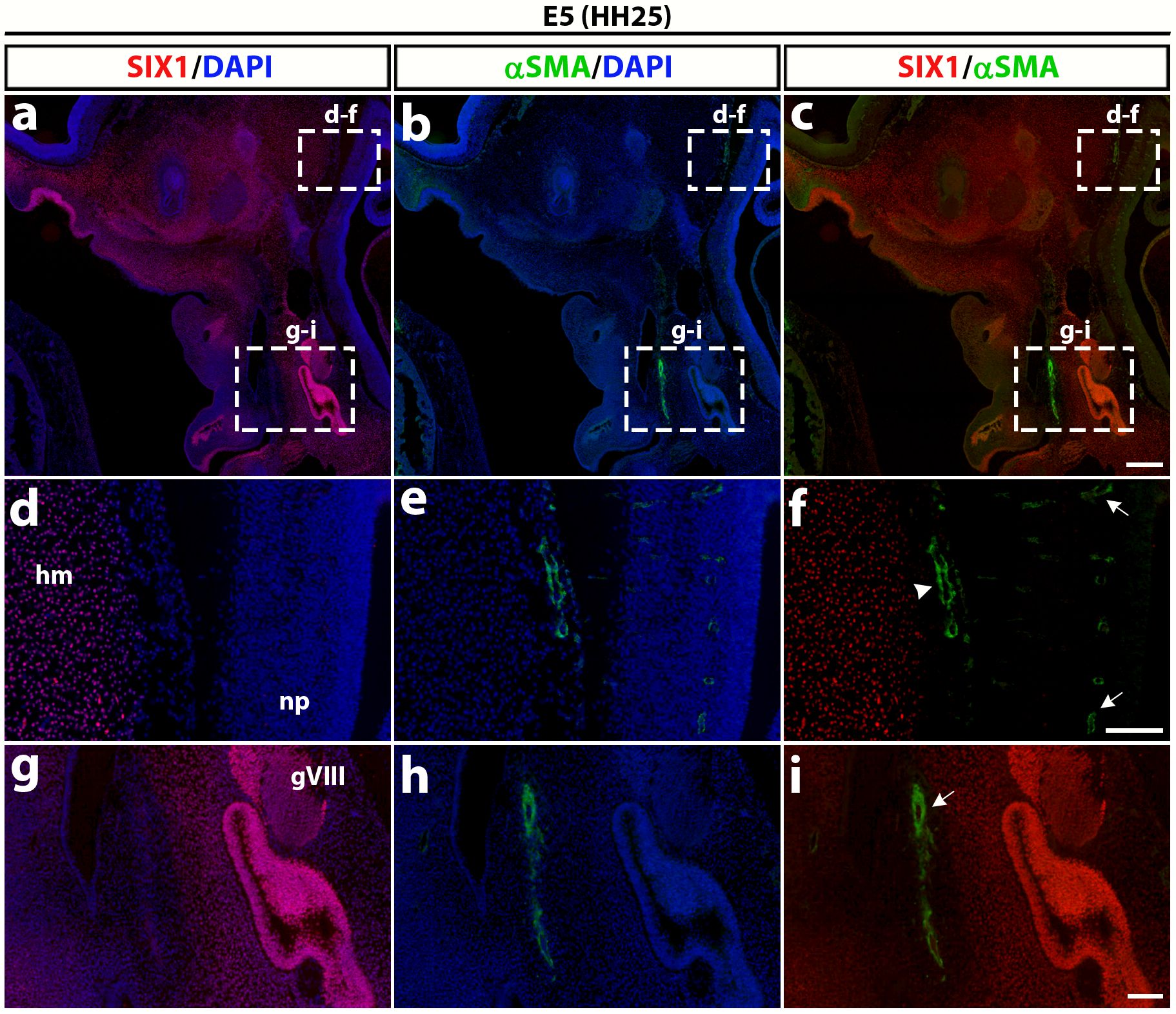

Supplement: Supplementary file 2 — Absence of SIX1-immunoreactive cells in head blood vessels at E5. (a, d, g) SIX1 immunostaining in sections of an E5 (HH25) chicken head, counterstained with DAPI. (b, e, h) αSMA immunostaining and DAPI. (c, f, i) Merge of SIX1 and αSMA labeling. (d-f) Magnifications of the area depicted in a-c; note SIX1 expression in head mesenchyme (hm in d) but not in the wall of blood vessels (arrowhead in f) adjacent to the neuroepithelium (np in d), neither in αSMA+ pericytes within the brain epithelium (arrows in f). g-i Magnifications of the area depicted in a-c; observe strong SIX1 staining (g) in the inner ear epithelium and associated vestibulo-acoustic ganglion (gVIII), whereas αSMA+ smooth muscle cells lining blood vessels lack SIX1 expression (arrow in i). Scale bars, 100 μm, except in a-c, 300 μm. (TIFF 3552 kb) [file 12861_2017_155_MOESM2_ESM.tif]
